# An integrative approach to the regulation of mitochondrial respiration during exercise: Focus on high-intensity exercise

**DOI:** 10.1016/j.redox.2020.101478

**Published:** 2020-02-25

**Authors:** Jose A.L. Calbet, Saúl Martín-Rodríguez, Marcos Martin-Rincon, David Morales-Alamo

**Affiliations:** a Department of Physical Education, University of Las Palmas de Gran Canaria, Campus Universitario de Tafira s/n, 35017, Las Palmas de Gran Canaria, Spain; b Research Institute of Biomedical and Health Sciences (IUIBS), University of Las Palmas de Gran Canaria, Paseo Blas Cabrera Felipe “Físico” (s/n), 35017, Las Palmas de Gran Canaria, Canary Islands, Spain; c Department of Physical Performance, The Norwegian School of Sport Sciences, Postboks, 4014 Ulleval Stadion, 0806 Oslo, Norway

**Keywords:** Mitochondrial respiration, High-intensity exercise, Oxidative stress, Sprint performance, Fatigue

## Abstract

During exercise, muscle ATP demand increases with intensity, and at the highest power output, ATP consumption may increase more than 100-fold above the resting level. The rate of mitochondrial ATP production during exercise depends on the availability of O_2_, carbon substrates, reducing equivalents, ADP, P_i_, free creatine, and Ca^2+^. It may also be modulated by acidosis, nitric oxide and reactive oxygen and nitrogen species (RONS). During fatiguing and repeated sprint exercise, RONS production may cause oxidative stress and damage to cellular structures and may reduce mitochondrial efficiency. Human studies indicate that the relatively low mitochondrial respiratory rates observed during sprint exercise are not due to lack of O_2_, or insufficient provision of Ca^2+^, reduced equivalents or carbon substrates, being a suboptimal stimulation by ADP the most plausible explanation. Recent *in vitro* studies with isolated skeletal muscle mitochondria, studied in conditions mimicking different exercise intensities, indicate that ROS production during aerobic exercise amounts to 1-2 orders of magnitude lower than previously thought. In this review, we will focus on the mechanisms regulating mitochondrial respiration, particularly during high-intensity exercise. We will analyze the factors that limit mitochondrial respiration and those that determine mitochondrial efficiency during exercise. Lastly, the differences in mitochondrial respiration between men and women will be addressed.

## Graphical abstract

**Figure:**
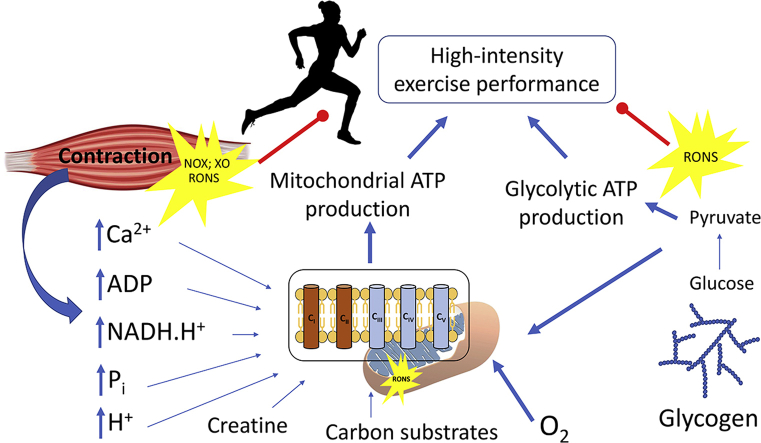

## Abbreviations

ΔΨ: Mitochondrial membrane potential
Acetyl-CoA: Acetyl coenzyme A
ADP: Adenosine diphosphate
ANT: Adenine nucleotide translocase
ATP: Adenosine triphosphate
ATP-Mg/P_i_ carriers: ATP-Mg/inorganic phosphate (P_i_) carriers
AMPK: AMP-activated protein kinase
AGC: aspartate/glutamate carriers
Ca^2+^: Ion calcium
CaMC: Ca^2+^ binding mitochondrial carriers
CAMKKβ: Ca^2+^/calmodulin-dependent protein kinase kinase-β
CaMKII: Calmodulin-dependent Protein Kinase II
CD36: fatty acid transporter CD36
CK: Creatine kinase
COX: Cytochrome C Oxidase
Cr: Creatine
eNOS: endothelial nitric oxide synthase
GSSG: Glutathione disulfide
H_2_PO_4_: Phosphoric acid
IMM: Inner mitochondrial membrane
IMS: Intermembrane mitochondrial space
MCU: Mitochondrial calcium uniporter
Mito_VD_: Mitochondrial volume density
MtCK: Mitochondrial creatine kinase
NAD^+^: Nicotinamide adenine dinucleotide oxidized
NADH^+^: Nicotinamide adenine dinucleotide reduced
nNOS: neuronal nitric oxide synthase
OXPHOS: Mitochondrial oxidative phosphorylation
OMM: Outer mitochondrial membrane
P_a_O_2_: Arterial O_2_ pressure
PCr: Phosphocreatine
PDH: Pyruvate dehydrogenase
PDHC: Pyruvate dehydrogenase complex
PFKFB3: 6-phosphofructo-2-kinase/fructose-2,6-bisphosphatase 3
P_i_: Inorganic phosphate
P_I_O_2_: Inspiratory O_2_ pressure
PO_2_: O_2_ pressure
RNS: Reactive nitrogen species
ROS: Reactive oxygen species
RONS: Reactive oxygen and nitrogen species
SERCA: Sarco/endoplasmic reticulum Ca^2+^-ATPase
TRX: Thioredoxin
TCA: Tricarboxylic acid cycle
VDAC: voltage-dependent anion channel (or porin)
VO_2_: Oxygen consumption
VO_2_max: Maximal oxygen consumption
VO_2_peak: Peak oxygen consumption

## 1 Introduction

Mitochondria originated nearly 2 billion years ago by the endosymbiosis of a specialized prokaryote [1]. The main function of mitochondria is the synthesis of ATP through oxidative phosphorylation, in which carbon substrates are oxidized with electrons flowing through the respiratory chain complexes to generate a proton gradient across the inner mitochondrial membrane (IMM). According to Mitchel's chemiosmotic theory [2], this proton gradient provides the energy necessary for the production of ATP. Besides, the proton gradient is the driving force for the mitochondrial uptake of positively charged ions and for the transport of ADP from the cytosol to the mitochondria [3]. During oxidative phosphorylation (OXPHOS), some reactive oxygen species (ROS), whose quantity in physiological conditions is 1-2 orders of magnitude lower than thought [5], are generated [4]. Although the rate of ROS production is low, oxidative stress has been detected during fatiguing and intense prolonged exercise [6]. Unchecked production of ROS and nitrogen reactive species (RNS) may be damaging [7] and could jeopardize cell survival [8]. The skeletal muscle and the mitochondria have redundant antioxidant systems and enzymes [6], with remarkable adaptive capacity to exercise training, which prevents oxidative damage by repeated exercise. ROS also play a critical signaling role, and partial counteraction of ROS production during exercise has been associated with incomplete adaptation to exercise training, blunting some of the beneficial adaptations of regular exercise [9,10]. In this review, we will focus on the mechanisms regulating mitochondrial respiration, particularly during high-intensity exercise. We will analyze the factors that limit mitochondrial respiration, with emphasis on the roles played by oxygen, reduced equivalents, carbon substrates, calcium, ROS, metabolites, and the factors that determine mitochondrial efficiency during exercise. Lastly, the differences in mitochondrial respiration between men and women will be addressed.

## 2 Regulation of mitochondrial respiration during exercise: some insights from sprint exercise in humans

During exercise, muscle ATP demand increases with exercise intensity and at the highest power output, ATP consumption may reach more than 100-fold the value observed at rest. ATP supply must match the ATP demand to maintain power output; otherwise power would progressively decline to a level at which it is possible to match energy demand and supply. These high rates of ATP output require a concerted contribution of both anaerobic (i.e., phosphocreatine and glycolysis), and aerobic ATP resynthesis mechanisms. At peak power output, as reached during maximal sprinting, the demand for ATP per second may exceed more than 10-fold the aerobic ATP resynthesis observed in the sprint [11]. Nonetheless, during a short sprint, the rate of O_2_ consumption is only 20–30% of VO_2_max [[11], [12], [13]]. Therefore, during maximal sprinting most of the ATP is resynthesized by the anaerobic metabolism, causing accumulation of end-products linked to muscle fatigue [14]. The strong activation of the anaerobic metabolism during exercise is connected to the production of reactive oxygen and nitrogen species (RONS), which may also contribute to muscle fatigue [15]. Aerobic ATP production results in the generation of H_2_O, CO_2_ and heat, as well as RONS [4] (see below RONS and mitochondrial respiration during exercise), with heat and RONS as potential contributors to fatigue. Heat accumulation is minimal during a short sprint in human skeletal muscle [16], and if anything, the moderate increase in muscle temperature may counteract fatigue [17]. The production of RONS is reduced with the increase in respiratory rate (O_2_ consumption, VO_2_), explained in part by lower accumulation of partially reduced intermediates capable of generating superoxide radicals, and due to a lower local oxygen availability in the microenvironment [3,18]. Thus, a lower RONS production from mitochondrial sources is expected as the VO_2_ increases during the sprint [16]. Production of RONS by mitochondrial dehydrogenases is stimulated by high NADH/NAD^+^ levels and inhibited by NAD^+^. The rate of NAD^+^ output is enhanced by high respiratory rates [3,[19], [20], [21]]. ADP plays a critical role in the regulation of energy metabolism during exercise since it regulates the rate of both the glycolytic flux [22] and OXPHOS [23].

### 2.1 Why do mitochondria do not respire at a higher rate during sprint exercise?

During a short sprint, the mitochondria are respiring at submaximal intensity. The question is then why mitochondria do not respire at a higher rate. We tried to answer this question by examining the different steps of the O_2_ cascade from the atmosphere to the skeletal muscles [16]. The energy necessary for the synthesis of ATP in the mitochondria is provided by the proton gradient across the inner mitochondrial membrane. This gradient is generated through electron transport reactions that are linked to the oxidation of carbon substrates. The rate of mitochondrial ATP production depends on the availability of O_2_, carbon substrates, reducing equivalents, ADP, P_i_, Ca^2+^, and free creatine (Cr), as well as the stimulation by ADP [24].

### 2.2 Is oxygen delivery a limiting factor for mitochondrial respiration during sprint exercise in humans?

**Fig. 1:**
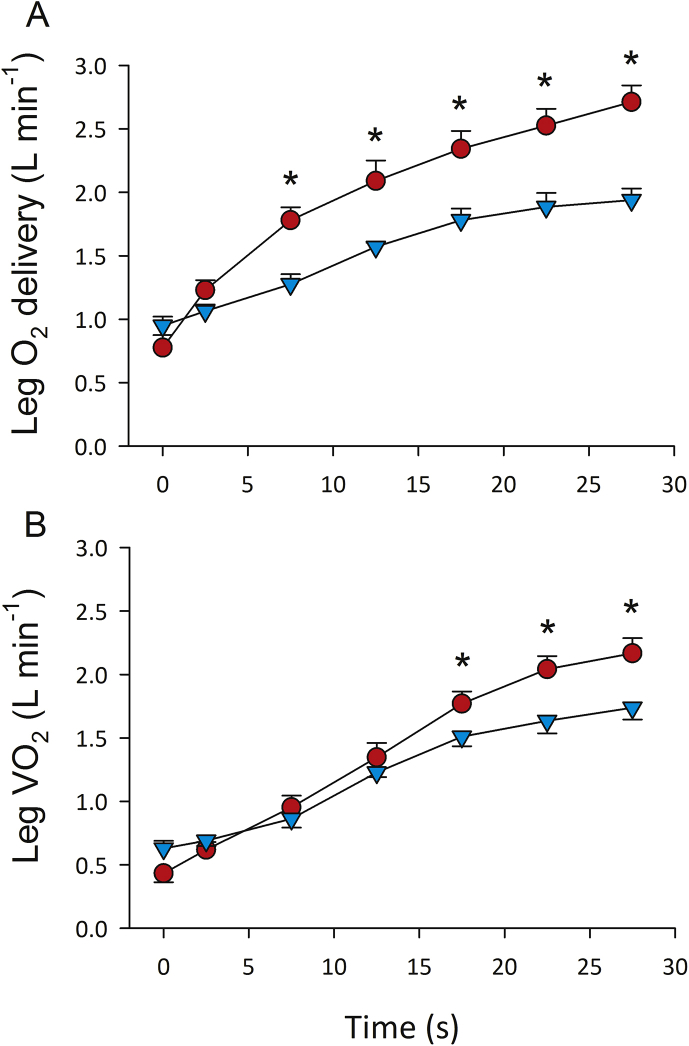
**Oxygen delivery (A) and consumption (VO**_**2**_**) (B) during sprint exercise in men**. Oxygen delivery and VO_2_ were measured by the direct Fick method during 30-s all out sprints in normoxia (red circles) and severe acute hypoxia equivalent an altitude of 5300 m above sea level (light blue circles; P_I_O_2_ = 73 mmHg). The symbol (*) indicates significant differences between normoxia and hypoxia. Leg VO_2_ was similar in both conditions despite large differences in O_2_ delivery, indicating that at least during the first 15 s O_2_ delivery was not limiting mitochondrial respiration when the exercise was carried out in normoxia (modified from Calbet et al. [16]). (For interpretation of the references to colour in this figure legend, the reader is referred to the Web version of this article.)

To determine why VO_2_ is so low despite a maximal ATP demand during sprint exercise, we asked some volunteers to perform maximal 10 or 30-s sprints in severe acute hypoxia (close to the level of human tolerance, P_I_O_2_ = 74 mmHg, PaO_2_ = 33 mmHg) and normoxia while measuring leg blood flow by thermodilution and the femoral's artery and vein O_2_ concentrations using the direct Fick method, which requires catheterization of the femoral artery and vein. During sprint exercise in normoxia and severe acute hypoxia, skeletal muscle VO_2_ is almost the same at the start of the sprint (first 10 s) and only slightly lower from the 15th to the 30th second, despite much higher oxygen delivery in normoxia (Fig. 1). These experiments demonstrated that muscle VO_2_ is not limited by O_2_ delivery during a maximal short sprint in normoxia [16]. This should not be surprising since *in vitro* experiments in cardiomyocytes have shown that mitochondria isolated in 20.9% O_2_ and 0.1% O_2_ both responded with significantly increased ATP levels to the stimulation of succinate and ADP, with greater production of ATP upon stimulation in the hypoxic isolated mitochondria compared to the normoxic isolated mitochondria [24]. Besides this, the Nanadikar et al. [24] experiment demonstrated that isolated mitochondria could operate at very low O_2_ tensions (i.e., PO_2_ < 1 mmHg).

### 2.3 Is the provision of reduced equivalents and substrates limiting OXPHOS during sprint exercise?

During sprint exercise, the production of reduced equivalents (NADH.H^+^) is also in excess due to the high glycolytic rate elicited by sprint exercise [[25], [26], [27]]. Moreover, during sprint exercise in severe acute hypoxia, NADH.H^+^ is markedly higher and NAD^+^ lower than during the same sprints performed in normoxia [[25], [26], [27]]. Although the glycolysis produces NADH.H^+^ in the sarcoplasm, it can be rapidly transferred to the mitochondrial matrix by the malate-aspartate NADH shuttle. Thus, the low mitochondrial respiratory rate observed during sprint exercise in normoxia does not seem to be due to a lack of NADH.H^+^.

Insufficient provision of pyruvate can also be ruled out since the pyruvate dehydrogenase (PDH) is completely dephosphorylated (activated) during sprint exercise, both in normoxia and severe acute hypoxia [25,27]. Moreover, there is a substantial increase in muscle lactate, also reflecting the high glycolytic rate and production of pyruvate [25,27,28]. In theory, acetyl group availability may be a limiting factor for OXPHOS at the start of the sprint [29]. Nevertheless, it has been suggested that acetyl group deficit may occur only at moderate exercise intensities (65–90% VO_2_max) [30]. At higher exercise intensities (90–110% VO_2_max), activation of PDH with dichloroacetate (an inhibitor of PDH kinase) did not influence exercise performance, nor did it influence O_2_ utilization, despite the fact that acetyl-CoA was increased before the start of exercise and after the administration of dichloroacetate. Similar results were reported during sprint exercise, i.e., the administration before sprint exercise of either acetate to increase resting acetyl-CoA and acetyl-carnitine, or dichloroacetate to increase resting acetyl-CoA, acetyl-carnitine and PDH activation, did not affect non-oxidative ATP production nor performance [28]. Although Howlett et al. [28] did not directly measure muscle VO_2_, the fact that the anaerobic energy production was not affected strongly suggests a similar VO_2_, indicating that acetyl group availability is not a limiting factor for OXPHOS during sprint exercise. These experiments showed that the provision of acetyl-CoA groups is not a limiting factor for substrate oxidation during high-intensity exercise in humans [28,[31], [32], [33]].

A deficit of other carbon substrates for OXPHOS, downstream to acetyl-CoA, such as tricarboxylic cycle (TCA) intermediates, has also been suggested as potential limiting factors for aerobic energy production, at least during prolonged exercise to exhaustion [34]. There is no information regarding the muscle concentration of TCA intermediates during sprint exercise in humans. Skeletal muscle TCA intermediates, particularly succinate, malate, and fumarate, increase during the first minute of moderate-intensity exercise [35]. Supplementation with citrulline malate before repeated sprint exercise did not affect sprint performance [36], suggesting that the availability of malate is not limiting. However, without the measurement of muscle malate concentrates or VO_2_, no conclusion can be made regarding the effect of malate availability on mitochondrial respiration. The fact that blood lactate concentration is not altered by citrulline malate could indicate that malate is not limiting OXPHOS during sprint exercise in humans.

### 2.4 Calcium regulation of OXPHOS during sprint exercise

Sarcoplasmic calcium concentration ([Ca^2+^]) is presumably maximal during sprint exercise; otherwise power output will not be maximal, and therefore insufficient availability of Ca^2+^ is unlikely. Calcium may stimulate mitochondrial respiration by multiple mechanisms enhancing energy consumption such as providing reduced equivalents and substrates for OXPHOS, increasing the activity of several dehydrogenases and modulating F1-FO-ATPase and cytochrome oxidase [37]. Calcium triggers muscle contraction while simultaneously upregulating glycogenolysis, glycolysis, and glucose transport. Sarcoplasmic Ca^2+^ activates calmodulin-dependent protein kinase II (CaMKII), which in turn stimulates glycogenolysis and glycolysis by activating several of the critical enzymes involved (phosphorylate aldolase A, glyceraldehyde-3-phosphate dehydrogenase, enolase, lactate dehydrogenase, creatine kinase, pyruvate kinase, and phosphorylase *b* kinase) [[38], [39], [40]]. Besides, Ca^2+^ promotes the synthesis of fructose 2,6‐bisphosphate (F2,6BP), which in turn activates phosphofructokinase (PFK). In skeletal muscle, the bifunctional enzyme responsible for F2,6BP formation is the 6-phosphofructo-2-kinase/fructose-2,6-bisphosphatase 3 (PFKFB3) [41], which is activated via phosphorylation by AMPK [42]. AMPK is phosphorylated and activated by the Ca^2+^ sensitive CAMKKβ, an upstream kinase for AMPK [43]. Blunting CaMKII activation during sprint exercise reduces the glycolytic rate slightly without significantly altering performance [26,44]. This suggests that the provision of reduced equivalents and glycolytic substrates is in excess during sprint exercise.

Sarcoplasmic Ca^2+^ may act directly on Ca^2+^ binding mitochondrial carriers (CaMC), which host a specific binding site located on the outer side of the inner mitochondrial membrane, i.e., without need to penetrate in the mitochondrial matrix [45]. The main two CaMCs are the aspartate/glutamate carriers (AGC) [46], also called aralar1 (AGC1) and the ATP-Mg/inorganic phosphate (P_i_) carriers (ATP-Mg/P_i_ carriers), also named SCaMC (for short CaMC) [47] (for review see Ref. [48]). Aralar is a member of the malate-aspartate NADH shuttle, present in skeletal muscle, which upon Ca^2+^ binding promotes the exchange of aspartate by glutamate and the transport of NADH into the matrix. Furthermore, an increase in mitochondrial glutamate stimulates state 3 respiration [49]. The malate-aspartate NADH shuttle activity is likely stimulated by small increases of sarcoplasmic Ca^2+^, as extramitochondrial Ca^2+^ does in the brain [50], i.e., below the Ca^2+^ concentrations at which the calcium uniporter (MCU) is activated. The role that AGC1 has on the activation of mitochondrial respiration at the start of intense exercise remains unknown.

Ca^2+^ stimulates oxidative phosphorylation by eliciting a drop in the ATP/ADP ratio through the stimulation of muscle contraction and SERCA, but also after its transport into the mitochondria through the MCU. Once in the matrix, Ca^2+^ activates pyruvate, isocitrate, and α-ketoglutarate dehydrogenases. The entry of Ca^2+^ may be facilitated by large increases of [Ca^2+^] in the microenvironment between sarcoplasmic reticulum and mitochondria [[51], [52], [53]].

Mitochondrial Ca^2+^ influx is driven by the membrane potential (ΔΨ). Since Ca^2+^ carries two positive charges, the entry of Ca^2+^ in the mitochondria entails a reduction of the chemical component (ΔpH) of the electrochemical potential due to charge compensation by the extrusion of 2H^+^, alkalinizing the mitochondrial matrix [54]. Therefore, the entry of Ca^2+^ reduces the ΔΨ, limiting the driving force for the influx of more Ca^2+^, unless Ca^2+^ transport occurs accompanied by anions in the protonated form, neutralizing the pH gradient and regenerating ΔΨ [55]. The most important of these anions accompanying the influx of Ca^2+^ is H_3_PO_4_, which is transported into the mitochondrial matrix by the P_i_-H^+^ symporter [56], which likely plays a critical role in the stimulation of mitochondrial respiration at the start of exercise [57]. Ca^2+^ uptake and ADP phosphorylation compete for the electrochemical gradient generated by respiration [3]. Ca^2+^ uptake precedes ADP phosphorylation when they are simultaneously added to the reaction medium [58].

PDH activation can be used as a surrogate marker of matrix [Ca^2+^] [37]. Parolin et al. demonstrated that at 6th second of a 30-s all-out sprint (isokinetic Wingate test), the activation of PDH had increased from 14% at rest to 48%, to reach 95% activation at the 15th s [59]. Thus, the increase of Ca^2+^ in the matrix is fast during sprint exercise. Increased [Ca^2+^] may decrease the K_M_ for ADP, facilitating the stimulation of mitochondrial respiration, as shown in skinned cardiomyocytes [60].

### 2.5 Regulation of mitochondrial respiration by reactive oxygen and nitrogen species

Skeletal muscle generates mostly superoxide (O_2_•^-^) and nitric oxide (^•^NO), which after reaction with other compounds form additional ROS and RNS, such as hydrogen peroxide (H_2_O_2_), hydroxyl radicals (^•^OH), singlet oxygen (an electronically excited form of O_2_, highly reactive but is not a radical), nitric oxide (^•^NO), and peroxynitrite (ONOO^−^, a strong oxidizing agent product of the reaction between superoxide and ^•^NO) [61]. Eleven sites that produce O_2_·^-^ and hydrogen peroxide H_2_O_2_ have been identified in mammalian mitochondria [62]. The contributing sites and the rate of ROS production vary with the type of substrate oxidized, the metabolic rate, and the nutritional state (caloric restriction, overfeeding, etc.) [62]. It remains unknown which sites are the primary producers of ROS during exercise *in vivo* [5].

**Fig. 2:**
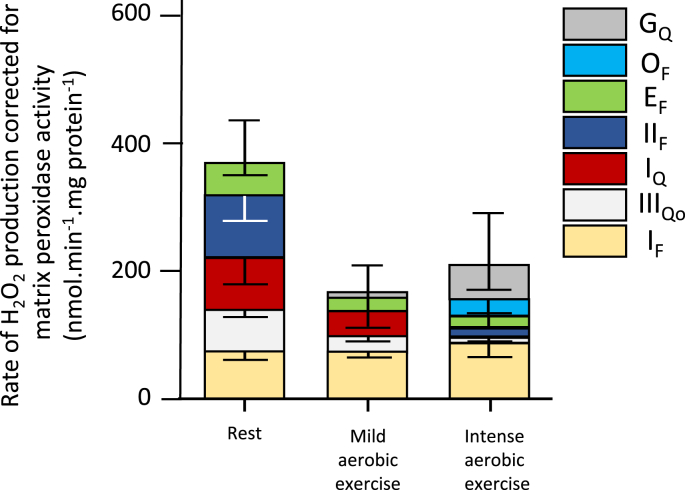
**Quantification of the relative contribution of several mitochondrial sites to superoxide and H**_**2**_**O**_**2**_**production in isolated rat skeletal muscle mitochondria assessed in a media mimicking conditions of rest, mild aerobic exercise, and intense aerobic exercise.** The authors applied corrections for H_2_O_2_ losses due to degradation by mitochondrial matrix peroxidases. Error bars in inverted position denote the propagated errors for each site while regular error bars indicate the propagated sum of these errors. Values are means ± S.E. (error bars) (n = 3–20). Modified from Goncalves et al. [5].

While at rest most ROS originate from the mitochondria, during intense exercise, the sarcoplasmic reticulum and transverse tubules or plasma membrane NAD(P)H oxidase (NOX) and, to a lesser extent, xanthine oxidase (XO) are considered the main sources [6,61,63]. During aerobic exercise mitochondrial ROS production is reduced [64] because skeletal muscle mitochondria are predominantly in state 3 respiration (ADP-stimulated) [65]. Gonsalves et al. [5] performed *in vitro* experiments with isolated skeletal muscle mitochondria reproducing physiological concentrations of substrates and effectors concentrations mimicking rest (10% VO_2_max), and low (22% VO_2_max) and intense (90% VO_2_max) aerobic exercise intensities. Electron leak was 0.35, 0.03, and 0.01% at 10, 22, and 90% of VO_2_max-simulated conditions, indicating that during exercise the mitochondria barely contribute to producing ROS, with a predominance of ROS originating in Complex I (site I_F_) during the “simulated” aerobic exercise [5] (Fig. 2).

Based on a complex model of muscle and whole-body metabolism in humans, Nilsson et al. [66] have proposed a bypass of the complex I during high-intensity exercise, as a mechanism to increase the catalytic capacity of the muscle (ATP production) at the expense of substrate efficiency. The model predicts that to bypass complex I, NADH is re-routed through the glycerol-3-phosphate shuttle, which transfers the NADH electrons directly to ubiquinone (QH_2_), entering the electron transport chain at complex III. Recent studies have shown that the proton-pumping step of complex I can be bypassed *in vivo* while retaining the NADH dehydrogenase activity [67]. This disengagement of the proton-pumping step of complex I is explained by a slow transition of the active (A) to the deactivated, dormant (D) form enzyme, as observed during ischemia in metabolically active organs such as the heart and brain [68]. With re-oxygenation, complex I reactivates increasing the production of ROS [68]. When the enzyme is in the D form is more susceptible to oxidation by reactive nitrogen species, resulting in nitrosylation of the enzyme. The latter has been proposed as an intrinsic protective mechanism that delays activation upon re-oxygenation, decreasing the production of ROS at the beginning of reperfusion [68]. It remains unknown, however, whether complex I can be bypassed during sprint exercise in human skeletal muscle. This could be the case with repeated prolonged sprints associated with oxidative stress [69,70], or when ischemia is applied at exhaustion followed after a few seconds by another bout of exercise with reperfusion [11,71].

As fatigue develops more RONS are produced, particularly if concurrent with the recruitment of anaerobic metabolism [6]. The fact that the high energy turnover achieved during sprint exercise elicits a greater production of ROS compared to low-intensity exercise or resting is supported by the observation of increased oxidative stress, the activation of ROS-induced signaling and the physiological adaptations increasing the antioxidant enzymes [61,69].

Current experimental evidence indicates that during fatiguing repeated sprint exercise, RONS production may cause oxidative stress and damage to cellular structures [69,70]. Therefore, to some extent, RONS-elicited fatigue [15] may contribute to avoid damage by ROS.

#### 2.5.1 Pyruvate dehydrogenase complex is a source of ROS during sprint exercise

The pyruvate dehydrogenases complex (PDHC) catalyzes the irreversible conversion of pyruvate to acetyl-CoA [72]. PDHC is an important source of H_2_O_2_ in experiments with permeabilized human muscle fibers [73]. When pyruvate and NAD^+^ are abundant, PDHC produces H_2_O_2_ at a low rate. Nevertheless, when the NAD^+^/NADH^+^ ratio is lowered, the PDHC reactions release more H_2_O_2_ [73]. The H_2_O_2_ generated by PDHC is converted to H_2_O by reduced thioredoxin (TRx), while TRx becomes oxidized. Oxidized TRx is immediately reduced by glutathione disulfide (GSSG) through the glutathione reaction using NADPH^+^ as a cofactor (producing NADP^+^). NADPH^+^ should be resynthesized by nicotinamide nucleotide transhydrogenase (NNT) with consumption of NADH^+^ as shown the following reaction [74]:

NADH^+^ + NADP^+^ ⇐ NNT ⇒ NAD^+^ + NADPH^+^

The latter agrees with the attenuation of the sprint exercise-elicited reduction of the NAD^+^/NADH^+^ ratio by the ingestion of an antioxidant cocktail (vitamin C, vitamin E, and alpha-lipoic acid) before de exercise [44]. In the latter study, the VO_2_ and the power output were similar in the sprints performed with placebo and antioxidants, while lactate accumulation was reduced by the ingestion of antioxidants [27,44]. These results indicate enhanced work efficiency during the sprints performed after the intake of antioxidants.

### 2.7 Role of metabolite accumulation in the regulation of mitochondrial respiration during sprint exercise

We hypothesized that during short sprints, the mitochondria do not generate more ATP, likely due to insufficient stimulation of the respiratory rate [11,16]. Some exercise is required for ADP and P_i_ to increase and accumulate to stimulate mitochondrial respiration [33]. However, during 30-s all-out sprints metabolite accumulation, including ADP, pyruvate, lactate, H^+^, and P_i_ approaches maximal levels [11,75]. Sarcoplasmic ADP should be transported to the mitochondrial matrix in a process linked to the proton gradient. Although adenine nucleotide transport is rapid, it may be the rate-limiting step in cellular respiration [76].

Despite the abundance of ADP, VO_2_ only reaches 80% of VO_2_max during the sprint [13,25,26]. Besides, the high glycolytic rate reached during sprint exercise [25,59,77] and the subsequent acidification of the muscle fibers could limit OXPHOS [78,79].

Acidification is expected to reduce the glycolytic rate by slowing glycogenolysis at the level of the glycogen phosphorylase *a* (active form) and glycolysis at the level of phosphofructokinase [80]. Although lactate is a potent antioxidant at physiological pH, due to its scavenging activity toward both superoxide and ^•^OH [81], the accompanying H^+^ accelerates the rate of dismutation of O_2^−^_^•^ to H_2_O_2_, which can react with Fe^2+^ to produce ^•^OH. In addition, acidosis facilitates the conversion of O_2^−^_^•^ to the more reactive and more liposoluble hydroperoxyl radical HO_2_^•^ and facilitates the dissociation of protein-bound iron [79] (iron is also a strong oxidant). Nevertheless, the administration of antioxidants has no effect on VO_2_ during high-intensity exercise [82].

To find out whether acidosis and metabolite accumulation limits OXPHOS we asked some volunteers to perform an incremental exercise to exhaustion. Right at exhaustion, we occluded the circulation of both legs during 10-s or 60-s in separate trials. At the end of the occlusion period, the circulation was re-established, and the subjects performed a 10-s all-out sprint, with VO_2_ reaching values 2 to 3-fold higher in these two sprints than observed in a control sprint in the rested condition [11]. During the occlusion, muscle O_2_ stores were depleted, phosphocreatine (PCr) further reduced, while the glycolysis remained active eliciting a progressive accumulation of lactate and further acidification [11]. Actually, after 1 min of occlusion, muscle lactate was increased by 25% and pH further reduced. Despite the increased acidification, VO_2_ was much larger than observed when the sprint was performed in optimal conditions, i.e., 5 min after a standardized warm-up [11]. In follow-up experiments, we have seen that VO_2_max can be reached when supramaximal exercise is performed right after a cycle of exhaustion followed by 10–60 s of occlusion of the circulation. This indicates that acidosis and sarcoplasmic P_i_ accumulation to levels above those that can be reached at exhaustion under physiological conditions, do not prevent the attainment of VO_2_max [71,83]. Consequently, the potential *in vivo* inhibitory effect of acidosis or sarcoplasmic P_i_ accumulation on OXPHOS in skeletal is likely counteracted by other mechanisms such as increased ADP stimulation and increased muscle temperature.

Moreover, the combination of fatiguing sprinting exercise followed by occlusion and reperfusion is expected to cause substantial RONS production [71,83]. Nevertheless, even in these conditions, skeletal muscles can reach VO_2_max. Therefore, these experiments in humans demonstrate that OXPHOS is not inhibited by the level of acidification, P_i_ accumulation, and RONS production seen in working human muscles, or at least the inhibition caused is not sufficient to negatively influence VO_2_max. This is possible because the human skeletal muscle has a large functional reserve in mitochondrial respiratory capacity [84] or other mechanisms compensate for the potential adverse effects of acidosis, P_i_, and RONS.

### 2.8 Role of ADP transport from the sarcoplasm to the mitochondrial matrix

**Fig. 3:**
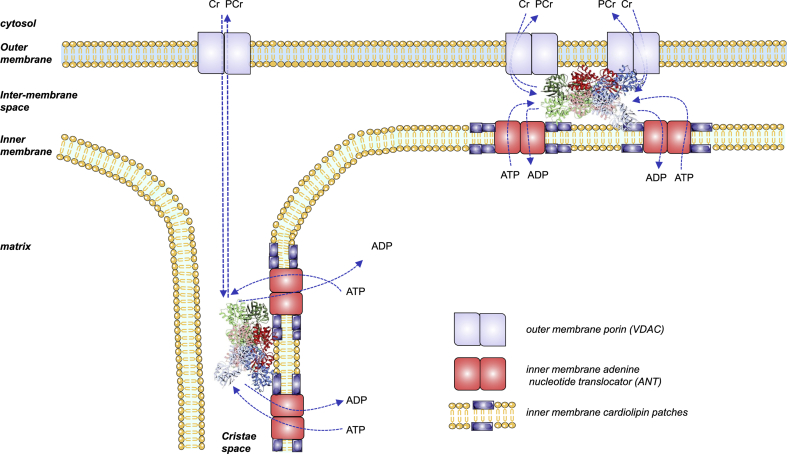
**ADP/ATP exchange in the mitochondria**. Mitochondrial creatine kinase (MtCK) forms proteolipid complexes with VDAC and ANT (‘‘contact site complexes’’, top right) or with ANT alone (‘‘cristae complexes’’, bottom left). ADP and ATP tend to accumulate in the intermembrane mitochondrial space (IMS) of the cristae in the vicinity of MtCK, facilitating the rapid resynthesis of ATP. The ATP flowing through ANT is rapidly hydrolyzed to ADP, which is sent back to the matrix by ANT. MtCK catalyzes the synthesis of PCr with the ATP provided by ANT, using the P_i_ and Cr available in IMS. The resulting PCr is exported to the sarcoplasm by VDAC. Modified from Schlattner et al. [96].

ADP, being the substrate for the synthesis of ATP, stimulates both the glycolysis [22] and OXPHOS [23]. Therefore, the transport of ADP from the sarcoplasm to the mitochondrial matrix is likely to determine the relative contribution of glycolytic and aerobic energy pathways to the resynthesis of ATP during high-intensity exercise. Three carrier protein complexes transport ADP from the sarcoplasm to the mitochondrial matrix: 1) the voltage-dependent anion channel (VDAC or porin) located on the outer mitochondrial membrane (OMM) [85]; 2) the mitochondrial creatine kinase (MtCK) [85]; and the adenine nucleotide translocase (ANT) [[86], [87], [88]] localized in cristae and intermembrane space [89]. MtCK increases the concentration of ADP within the intermembrane mitochondrial space (IMS) [90], where it is exchanged with ATP by the ANT. Since the OMM has a high permeability to PCr and Cr [91], both become rapidly available to the MtCK, which can use the ATP received from the ANT and the Cr diffusing from the sarcoplasm [92,93]. The rate of Cr and P_i_ supply is maximal during all-out sprints, however PCr is depleted within a few seconds after the start of the sprint [75]. This is due to the limitation imposed by the rate of ATP supply by the ANT since during high glycolytic rates, or when O_2_ is not available, there is no anaerobic resynthesis of PCr [11,94,95]. Due to spatial constraints, ADP and ATP tend to accumulate in IMS of the cristae in the vicinity of MtCK, facilitating the rapid resynthesis of ATP [96]. The ATP flowing through ANT is rapidly hydrolyzed to ADP, which is introduced in the matrix by ANT. At the same time, MtCK catalyzes the synthesis of PCr with the ATP provided by ANT, using the P_i_ and Cr available in the IMS. The resulting PCr is exported to the sarcoplasm by VDAC (Fig. 3).

VDAC forms an unspecific channel whose permeability is regulated by Ca^2+^ [97] and ROS [98,99]. VDAC can be found in contact sites between the inner and outer mitochondrial membranes forming proteolipid complexes containing adenine nucleotide translocase in the IMM, and VDAC in the OMM [100], which also can recruit additional proteins such as kinases that preferentially use mitochondrial ATP, including MtCK [96]. The interactions between MtCK and VDAC are stimulated by Ca^2+^, which interacts with VDAC in OMM [97], facilitating metabolite channeling and energy provision when sarcoplasmic Ca^2+^ increases, as during high-intensity exercise. Mitochondrial creatine kinase is particularly sensitive to oxidative damage, resulting in an impairment of metabolite channeling through the creatine phosphate shuttle [101]. The latter could, in theory, limit the aerobic contribution to the overall energy production [102]. Although the physiological relevance of this mechanism during exercise remains unknown, it is clinically meaningful, because ischemic preconditioning exerts its protective effects by preserving mitochondrial function and functional coupling between adenine nucleotide translocase and creatine kinase [103,104].

ANT activity can be regulated through tyrosine phosphorylation [105], lysine acetylation [106], glutathionylation [107], carbonylation [108], and nitrosylation [99], although the role of these modifications *in vivo* has not been established. For example, ANT acetylation is reduced after high-intensity exercise, which may facilitate the exchange flux of ADP/ATP [106]. Thus, most scientific evidence supports a crucial role of ANT in regulating mitochondrial respiration during exercise, and especially during sprint exercise, by establishing the flux rate of ADP from the sarcoplasm to the matrix.

## 3 Regulation of mitochondrial efficiency

Mitochondrial efficiency is defined here as the P/O ratio (ATP produced per O_2_ atom reduced by the respiratory chain), whose value may vary between tissues and whose calculation is extremely complex and prone to methodological errors [109]. The P/O ratio may be reduced by proton leak, proton slip, energy expended in substrate transport, and physiological mechanisms of uncoupling (see Hinkle et al. [109] for a review). The only substrates that use the ΔΨ during mitochondrial uptake are ADP plus P_i_ (in exchange for ATP) and glutamate (in exchange for aspartate) [109]. The single supplement for which there is experimental evidence indicating an improvement of the P/O ratio in humans is supplementation with nitrate, which is reduced to nitrite by commensal bacteria in the oral cavity [110]. Nitrite is then absorbed and further reduced to nitric oxide by nitrite reductases [111].

In skeletal muscle, NO is produced by nitric oxide synthases (NOS) that catalyze the conversion of l-arginine to l-citrulline and have an essential role in exercise-induced muscle signaling [112] and fatigue [113]. Neuronal (nNOS or NOS-1) and endothelial (eNOS or NOS-3) NOS are activated by the interaction of Ca^2+^ with calmodulin, and the expression of both NOS enzymes is upregulated by exercise training [[114], [116], [115]]. Nitric oxide may cause reversible inhibition of cytochrome *c* oxidase [117,118]. However, the physiological relevance *in vivo* remains unclear [119], since humans reach similar whole-body VO_2_peak values and performance during supramaximal exercise (105% VO_2_max) after the infusion of -L-NAME (a NOS inhibitor) [120].

Dietary nitrates, abundant in leafy vegetables, have been shown to increase the P/O ratio during state 4 respiration in isolated mitochondria, as well as the maximal rate of ATP production in human skeletal muscle fibers, although without effects during state 3 respiration [111]. Larsen et al. tested the same subjects during submaximal exercise and found a 3% lower VO_2_ (from 1.95 to 1.89 mL min^−1^) after three days on nitrate supplementation. Nevertheless, about one-third of the effects on VO_2_ could be attributed to a shift in substrate oxidation to carbohydrates, since RER was increased (placebo 0.883 ± 0.01 versus nitrate 0.914 ± 0.01) [111]. The improved mitochondrial efficiency was explained by a reduced leakage or slippage of protons across the inner mitochondrial membrane in part due to the reduced expression of ANT, which is considered a significant contributor to proton leak [87]. Nitrate supplementation has also been reported to reduce VO_2_max by 2.7% [121], an effect that could be explained by the inhibitory actions of NO on cytochrome *c* [117,118].

### 3.1 Cytochrome C oxidase (COX) and mitochondrial efficiency

Cytochrome C Oxidase (COX), also called Complex IV, is a transmembrane protein complex of the inner mitochondrial membrane [122]. It is the last step in the electron transport chain, and its principal function is to catalyze the reduction of O_2_ to water. Every COX protein is composed of fourteen subunits that combine differently to conform distinct isoforms of the enzyme [123], whose distribution has tissue specificity. COX IV-1 is ubiquitously expressed and is regulated by allosteric inhibition by a high ATP/ADP ratio [123]. COX IV-2 is expressed mainly in the lungs, fetal skeletal muscle, and neurons [123]. COX IV-2 expression decreases cell respiration and enhances metabolic efficiency [124]. Overexpression of COX IV-2 in primary human myotubes reduces respiration and H_2_O_2_ production [124]. In humans, COX IV-2 protein expression in mitochondrial skeletal muscle correlates negatively with the resting metabolic rate [124]. Little is known regarding the effects that training may have on the regulation of COX isoforms.

## 4 Sexual dysmorphism in mitochondrial respiratory control

In resting conditions and during the post-absorptive state, women tend to incorporate non-esterified fatty acids (NEFAs) into triacylglycerols to store fat. In contrast, men are more prone to produce energy through plasma NEFA oxidation [125]. As a consequence, women display increased intramyocellular lipid content [126]. During exercise, estrogens promote fat oxidation [127], and at the same relative exercise intensity, women obtain a higher fraction of the energy from the oxidation of fatty acids than men [[128], [129], [130], [131]]. Several structural, functional, and endocrine factors explain this sexual dimorphism in energy metabolism [125,132,133]. In addition to the higher circulating estrogens, women have higher serum leptin concentrations and more leptin receptors in their skeletal muscles [134] and higher insulin sensitivity [125].

A recent study using permeabilized fibers from eight men and women has found that women have similar maximal respiration rates and abundance of ANT, but lower mitochondrial ADP sensitivity (+30% apparent Km) and absolute respiration rates at a physiologically relevant ADP concentration (100 μM) than men [133]. A ~3-fold reduction of skeletal muscle ADP sensitivity has been observed in endurance trained humans [135], and by analogy the lower ADP sensitivity observed in women compared to men could be advantageous for prolonged exercise. Miotto et al. [133] found higher sensitivity to malonyl-CoA-mediated respiratory inhibition and more abundance of the fatty acid transporter CD36 in women. At the same time, no sex differences in carnitine palmitoyl transferase-I protein content- and palmitoyl-CoA-supported respiration were observed.

**Fig. 4:**
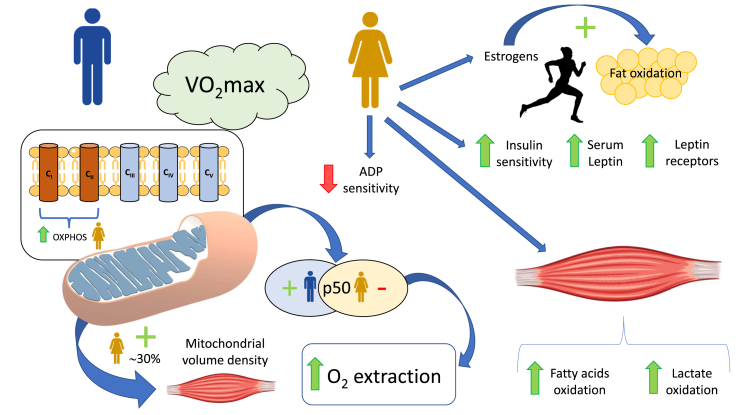
**Schematic representation of sexual dysmorphisms in mitochondrial function**. Higher circulating estrogens promote a greater contribution from fat oxidation to the overall energy expenditure in women than in men at the same relative exercise intensity. Compared to men, women exhibit higher serum leptin concentrations, higher insulin sensitivity, and more leptin receptors in muscle, but less ADP sensitivity. Experiments with permeabilized fiber indicate that women skeletal muscle fibers have increased maximal capacity to oxidize fatty acids and lactate. Women display lower mitochondrial p50 (increased mitochondrial oxygen affinity), and greater mitochondrial volume density, respiratory capacity (OXPHOS) and enhanced O_2_ extraction capacity when compared with men matched by VO_2_max. ADP = adenosine diphosphate; O_2_ = oxygen; OXPHOS = mitochondrial oxidative phosphorylation system; p50 = calculated oxygen pressure at 50% of the maximum flux; VO_2_max = maximal oxygen uptake.

In 12 men and 12 women matched by VO_2_max and exercise performance, a ~30% higher mitochondrial volume density (Mito_VD_) was observed in female *vastus lateralis* muscle [132]. The latter was accompanied by increased whole-body fat utilization during submaximal exercise compared with males, which was associated with an increased oxidative capacity for fatty acids and lactate in the permeabilized fibers. However, these differences in whole-body fat oxidation and skeletal muscle variables disappeared when adjusted for body mass or leg mass, which were lower in females [132]. More recently, Cardinale et al. [136] found higher C_I_, C_II_, and OXPHOS (C_I_ + C_II_) respiratory capacity (normalized to the amount of mitochondrial protein content) in women than men also matched by VO_2_max. In the same study, women displayed lower mitochondrial p50 (higher oxygen affinity) than men [136], which has been associated with enhanced O_2_ extraction capacity [137] (Fig. 4).

## 5 Conclusions

At low exercise intensities, ATP demand is matched by aerobic ATP resynthesis via oxidative phosphorylation. However, during sprint exercise, muscle ATP demand may increase more than 100-fold the resting level, exceeding the maximal *in vivo* mitochondrial ATP production by 2-3-fold in humans. The rate of mitochondrial respiration depends on the availability of O_2_, carbon substrates, reducing equivalents, ADP, P_i_ and Ca^2+^, and may be modulated by acidosis, nitric oxide, and reactive oxygen and nitrogen species. The relatively low mitochondrial respiratory rates observed during sprint exercise in humans are likely due to suboptimal stimulation by ADP, linked to insufficient ADP/ATP ANT antiport activity. Recent *in vitro* studies with isolated skeletal muscle mitochondria tested in conditions mimicking different exercise intensities indicate that ROS production during aerobic exercise is 1-2 orders of magnitude lower than previously thought. Mitochondrial efficiency is impaired by ROS and improved by nitrate supplementation, through the reduction of leakage or slippage of protons across the inner mitochondrial membrane. The latter has been attributed to a lower expression of the adenine nucleotide translocase and some reversible inhibition of cytochrome *c* oxidase. This may cause a small reduction (2–3%) of submaximal and maximal VO_2_. A sexual dimorphism exists in mitochondrial respiratory control with women displaying larger mitochondrial volume density, higher capacity to oxidize fat and sensitivity to malonyl-CoA-mediated respiratory inhibition, more abundance of the fatty acid transporter CD36, and lower mitochondrial ADP sensitivity. The mitochondrial p50 is also lower (increased affinity for O_2_) in women contributing to an enlarged O_2_ extraction capacity compared to men.

## Disclosure summary

The authors have nothing to disclose.

## Declaration of competing interest

The authors declare that they have no competing financial interests or personal relationships that could have appeared to influence the work reported in this paper.
